# The impact of periconceptional folate on the DNA methylome of acute lymphoblastic leukemia

**DOI:** 10.1038/s41375-026-02866-w

**Published:** 2026-03-24

**Authors:** Eric M. Nickels, Libby M. Morimoto, Alice Y. Kang, Catherine Metayer, Joseph L. Wiemels

**Affiliations:** 1 Cancer and Blood Disease Institute, Children’s Hospital Los Angeles, Los Angeles, CA USA; 2 Center for Genetic Epidemiology, Keck School of Medicine, University of Southern California, Los Angeles, CA USA; 3 Division of Epidemiology, School of Public Health, University of California, Berkeley, Berkeley, CA USA

**Keywords:** Cancer epidemiology, Acute lymphocytic leukaemia, Cancer prevention, Cancer epigenetics, Risk factors

## Abstract

Periconceptional folate intake decreases the risk of pediatric acute lymphoblastic leukemia (ALL); however, the mechanism is not fully understood. We sought to identify sites of DNA methylation measured at birth both responsive to periconceptional folate and associated with lymphoblasts at the time of ALL diagnosis in a “meet in the middle” analysis. Folate-associated differentially methylated regions (DMRs) were identified from retrospectively collected periconceptional maternal folate intake (by dietary source) and epigenome-wide DNA methylation status from archived dried neonatal blood spots for 189 ALL cases and 205 healthy matched controls from the California Childhood Leukemia Study (1995–2008). Folate and lymphoblast-associated DMRs overlapped at 17 sites related to total folate intake, 13 for folate from food, 10 for natural foods, 13 for fortified foods and 18 for folate-containing supplements. The majority of overlapping DMRs were in a concordant direction of effect for supplemental folate (16/18, *P* = 0.001). Opposite direction of effect was identified among lower income participants for food (3/19, *P* = 0.004) and natural folate (5/37, *P* < 0.001), the latter of which was specific to Hispanic participants of low-income (9/31, *P* = 0.029). These results indicate that dietary folate, in particular from natural food sources, may reduce risk of ALL through modulation of early DNA methylation patterns.

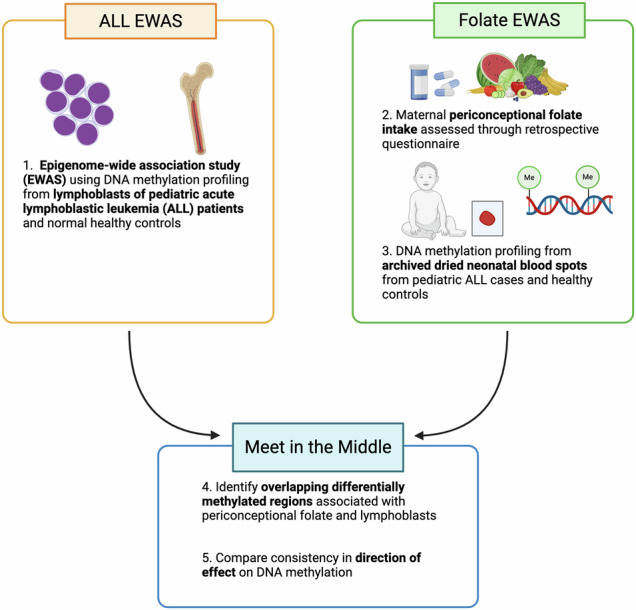

## Introduction

The etiology of pediatric acute lymphoblastic leukemia (ALL) is not fully understood; however, the disease is likely to arise in the intrauterine period via acquired genetic factors as influenced by early life environmental exposures and inherited genetic susceptibility risk factors [1–3]. Maternal nutrition [4, 5], including dietary [6] and supplemental [7, 8] intake of folate, is associated with reduced risk of ALL. Folate intake in the pre- and periconceptional periods [9] demonstrates greater inverse association with the development of childhood ALL than serum levels at birth [10], highlighting periconceptional development as a crucial timepoint influencing leukemia risk. While single nucleotide polymorphisms (SNP) in folate regulatory genes in children do not associate with ALL risk [11], interaction between maternal SNPs [12] and folate intake may influence this relationship [13]. While folate intake reduces ALL risk across all populations, children of Hispanic backgrounds [9, 14] and low education levels [15] specifically demonstrate enhanced risk-reducing benefits of maternal folate consumption.

DNA methylation is a stable, heritable marker largely established during embryogenesis and influenced by genetic, environmental and stochastic influences. Aberrant DNA methylation is a hallmark of pediatric ALL at diagnosis [16], while early alterations in DNA methylation patterns show a potential association with the future development of leukemia [17, 18]. Maternal folate intake in the periconceptional period was demonstrated to affect DNA methylation patterns in offspring [19, 20], providing a potential mechanism by which dietary intake of nutrients modulates development of ALL [21]. We previously showed that the influence of periconceptional folate on DNA methylation varies by the dietary and supplemental sources, as well as ethnicity and socioeconomic status [22]. We further identified variation in DNA methylation responsiveness to maternal folate in pediatric ALL cases compared to healthy controls. The manner in which folate-responsive DNA methylation sites may contribute to alteration in DNA methylation patterns at diagnosis is not fully understood. Prior work using a *meet-in-the-middle* approach [23] has shown consistency in the direction of effect at folate-responsive DNA methylation sites at birth and at diagnosis in pediatric ALL cases. Here, we employ a meet-in-the-middle approach to identify the relationship between early-life DNA methylation patterns responsive to maternal folate intake by dietary or supplemental source and alterations in methylation in leukemic blasts at the time of diagnosis using resources from the California Childhood Leukemia Study (CCLS).

## Materials/subjects and methods

### Participant and sample acquisition

Participants were identified from the CCLS, a population-based case-control study [24]. Cases were identified from children (age 0–14 years) diagnosed with leukemia at California hospitals between 1995 and 2008. Controls were randomly selected from birth certificates from the California Department of Public Health Office of Vital Records and matched by birth date (to match the date of diagnosis for cases), sex, maternal race, and Hispanic identity at a 1:1 or 2:1 ratio. Whole-genome DNA methylation analysis was conducted on a subset of cases with pediatric ALL and matched controls using the Illumina Infinium Methylation 450k or EPIC BeadChip platform [20, 25]. Dried blood spots (DBSs) were obtained from peripheral blood heel-stick samples from birth maintained by the California Department of Public Health. DNA was isolated from DBS samples and sent for DNA methylation analysis as described elsewhere [20].

Leukemic blasts were obtained from diagnostic bone marrow samples from CCLS cases with B-lineage ALL, while purified control B-cell samples (CD19+CD34+) were obtained from fetal bone marrow samples as described previously [26]. Set A included 227 leukemic blast samples obtained from bone marrow examination at the time of ALL diagnosis, along with CD19+CD34+ sorted pre-B-cells derived from 6 individual participants fetal bone marrow samples. Set B samples included 37 leukemic blast samples and 10 distinct CD19+CD34+ sorted pre-B-cells from fetal bone marrow controls. Mononuclear cell purification and genomic DNA isolation was completed as noted elsewhere [25]. A subset of participants underwent whole-genome DNA methylation profiling using the Illumina Infinium Methylation 450k array (Set A) or the EPIC BeadChip array (Set B).

### DNA methylation processing

Extracted DNA from DBS, leukemic blast and pre-B cell samples was modified by sodium bisulfite for conversion of unmethylated cytosines to uracil using the EZ DNA Methylation Kit (Zymo Research) for use in genome-wide methylation assessment. Samples were subsequently sent for methylation assessment using the Illumina Infinium Methylation EPIC BeadChip platform (Illumina) [20, 25]. Raw IDAT (intensity data file) files were imported into R (Version 4.0.0, The *R* Foundation) for data preprocessing and normalization using the *openSesame* pipeline from the *SeSAMe* package [27] in *minfi* [28] with the distribution of signal background calibrated by type I probe out-of-band signal. Probes with a detection *P*-value threshold >0.05 were masked from further analysis. *NOOB* background subtraction was performed, followed by removal of residual background. Nonlinear scaling was used to correct for dye balance. Probes and participants with >5% missing values were removed. Missing values were imputed using the *impute.knn* function in the *R* package *impute*. Chromosome X and Y probes were included in analysis.

### Folate quantification

Retrospective maternal dietary history was obtained by a modified Block food-frequency questionnaire (mBFFQ) [29] as described elsewhere [9, 22, 30]. Briefly, the mBFFQ surveyed the frequency of intake for common foods and supplements in the year prior to pregnancy and was administered at the time of diagnosis or enrollment for controls. A Spanish language version of the mBFFQ which included seven additional Hispanic-specific foods was offered. Folate intake was quantified as dietary folate equivalents (DFEs) as a total amount of intake, from food sources only (including both fortified and naturally occurring dietary folate sources), and from supplemental folic acid sources only such as multi-vitamins and pre-natal vitamins. Supplemental DFEs were obtained from the exact listed amounts on folic acid and multivitamin supplements. DFE measurements accounted for folic acid fortification in grain products starting in 1998 in the United States.

### Data analysis and statistical approach

DNA methylation beta values ranging from 0 (fully unmethylated) to 1 (fully methylated) were logit-transformed to M-values for statistical modeling and analysis. To identify probes significantly associated with periconceptional folate intake, multivariable linear regression was conducted independently for five folate sources (total, food, fortified, natural, and supplemental DFEs) controlling for array batch effect, ALL case status, sex, the top 10 principal components (PCs) for nucleated cell proportions and top 10 PCs for genetic ancestry. Reference-Free Adjustment for Cell Type Composition (ReFACTor) was used to estimate nucleated cell proportions [31], whereas genetic ancestry was estimated using the EPISTRUCTURE [32] in GLINT [33]. Supplemental DFEs were treated categorically (0, 194, 360, and 540 DFEs), whereas the remainder were treated as continuous variables. To identify probes significantly associated with leukemic blasts at the time of ALL diagnosis, multivariable linear regression was again employed independently for Set A samples (450k array) and Set B samples (EPIC array) controlling for ALL subtype and batch effect. Meta-analysis of the Set A and Set B linear regression results was conducted using METAL [34]. Differentially methylated regions (DMRs) were identified through *Comb-p* [35] using a seed *P* value < 0.05, maximum probe distance of 1000 base pairs, a minimum of 2 probes pe region, and a significance threshold of Šidák-corrected *P* value < 0.05 for both the folate regression analysis (individually by the 5 folate sources) and for the leukemia meta-analysis. Gene set enrichment analysis of overlapping regions for gene ontology and KEGG pathway terms was conducted using the *missMethyl* [36] package in R. Overlapping regions were identified using the genomic coordinates of DMRs identified in *Comb-p*. The direction of effect in each region was obtained by averaging regression coefficients for all probes contained within the DMR. Significant concurrent direction of effect was assessed by a binomial test using the number of concurrent and total overlapping regions. Subgroup analysis was conducted by Hispanic ethnicity of child and household annual income, defined as low (<$75 000) and high (≥$75 000), as well as by both Hispanic ethnicity and income status. Analysis was not completed in participants of high-income status stratified by Hispanic ethnicity due to a low sample size in these groups.

## Results

### Leukemia associated CpGs

**Fig. 1:**
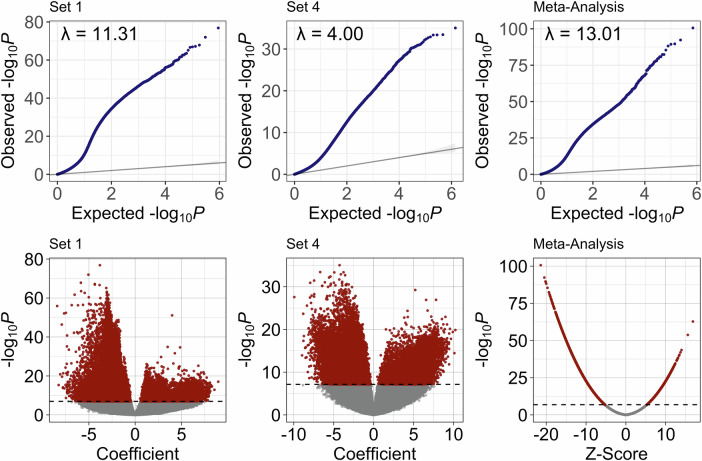
Lymphoblast-associated variation in DNA methylation. **A** Quantile-quantile plots of regression results comparing DNA methylation in lymphoblasts obtained at the time of ALL diagnosis and CD19+CD34+ pre-B cells. Genomic inflation values are shown for Set A (λ = 11.31), Set B (λ = 4.00) and for meta-analysis of both sets (λ = 13.01). **B** Volcano plots showing the distribution of *P*-values by regression coefficients or Z-scores in the case of meta-analysis results. A total of 69,906 CpGs met threshold for significance (*P* < 0.05) after Bonferroni correction for multiple comparisons for Set A, 27,728 CpGs for Set B, and 66,034 CpGs after meta-analysis of both datasets. Assessment of differentially methylated regions (DMRs) using Comb-P identified a total of 22,247 significant DMRs from meta-analysis results.

**Fig. 2:**
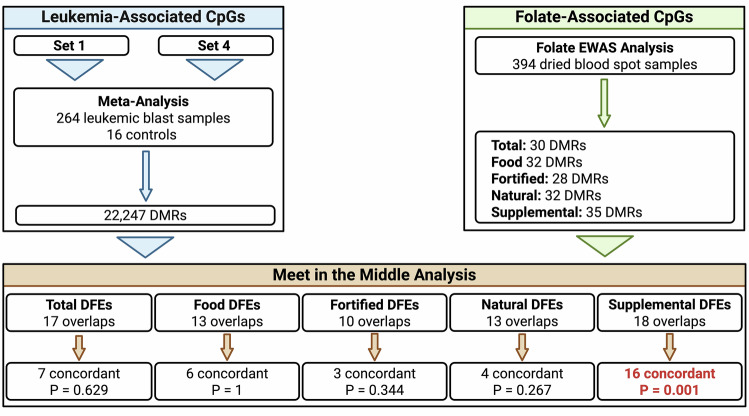
Overlapping differentially methylated regions. Differentially methylated regions (DMRs) were identified using Comb-P from two epigenome-wide association studies (EWAS) assessing 1) the relationship between periconceptional folate intake by source and DNA methylation at birth, including total folate (all sources), food folate (natural and fortified sources), and supplemental folic acid, and 2) DNA methylation variation in lymphoblasts at the time of pediatric acute lymphoblastic leukemia (ALL) diagnosis compared to healthy bone marrow or purified S2-phase pre-B lymphocytes. In the latter analysis, data from the California Childhood Leukemia Study (CCLS) were obtained at two separate timepoints (Set A and Set B) and subsequently meta-analyzed. To identify epigenomic regions with DNA methylation variation associated with both periconceptional folate at birth and lymphoblasts at the time of ALL diagnosis, DMRs from each study were subsequently assessed for overlapping genomic regions using a ‘Meet in the Middle’ approach. Overlapping DMRs were further analyzed to identify concordance in the direction of DNA methylation (hypermethylation vs hypomethylation). Binomial test of the concordant versus total overlapping regions identified a significant bias toward a concordant direction of effect in DMRs associated with supplemental folic acid (16 of 18 DMRs, *P* = 0.001).

Analysis of DNA methylation significantly associated with leukemic blasts was conducted in two subsets (Table 1). A total of 450,183 CpGs passed QC measures for analysis for Set A, and 700,796 for Set B. Multi-variate linear regression analysis was completed independently for both subsets controlling for ALL subtype and batch effect. In Set A, a total of 67,906 CpGs met threshold for significance (*P* < 0.05) after Bonferroni correction for multiple comparisons, while 27,728 CpGs were significant in Set B (Fig. 1). Meta-analysis of the linear regression results from Sets A and Set B was completed for 354,039 overlapping CpGs between the two arrays, resulting in 66,034 CpGs significantly associated with leukemic blasts after Bonferroni correction. This encompasses 60.1% of ALL-associated sites identified by Nordlund et al. (5652 of 9406 CpGs) [37]. The majority of these significant CpGs were hypomethylated in ALL samples (49,429, 74.9%) compared to hypermethylated (16,509, 25.1%). Genomic inflation λ for the three models were 11.31 for Set A, 4.00 for Set B, and 13.01 for the meta-analysis. To identify significant DMRs associated with leukemic blasts, the meta-analysis results were subsequently analyzed using *Comb-p*, resulting in 22 247 significant DMRs after Šidák correction for multiple comparisons (Fig. 2, Supplementary Table S1).

**Table 1:** Cohort characteristics.

Folate dataset	
Total subjects (N)	394
*Age at enrollment (years;median, IQR)*	4.6 (4.5)
*Male*	219
*Female*	175
*Case*	189
*Control*	205
Hispanic ethnicity	
*Hispanic*	175
*Non-Hispanic White*	160
Income status	
*Low-Income*	258
*High-Income*	137
*Hispanic, Low-Income*	150
*Hispanic, High-Income*	25
*Non-Hispanic White, High-Income*	78
*Non-Hispanic White, High-Income*	25
Leukemia dataset	
Set A	
*Leukemic blast samples*	227
*Bone marrow CD19*+*CD34+ pre-B cells*	6
Subtype	
*Hyperdiploid*	73
*t(12;21)*	52
*t(11;19)*	6
*MLL*	2
*Other*	73
*Unknown*	21
Set B	
*Leukemic blast samples*	37
*Fetal bone marrow controls*	10
Subtype	
*Hyperdiploid*	2
*t(12;21)*	11
*Unknown*	24

Folate dataset included acute lymphoblastic leukemia cases and controls from the California Childhood Leukemia Study (CCLS) with DNA methylation data from archived neonatal blood spots and survey-based periconceptional folate intake data. Leukemia dataset included pediatric ALL cases with DNA methylation data from lymphoblast samples at the time of leukemia diagnosis and CD34+CD19+ pre-B cells from healthy controls. Set A sample data was assessed using the Illumina 450k DNA methylation array, while Set B samples were assessed with the EPIC array.

*IQR* interquartile range.

### Folate-associated CpGs

A total of 394 participants with periconceptional folate intake survey data, including 189 ALL cases and 205 controls, underwent DNA methylation profiling from DBS samples obtained at birth using the EPIC array (Table 1). Mean folate intake by ALL case status and by demographic group are shown in Table 2. There was no significant difference between ALL cases and controls for total, food, fortified, and natural DFE categories. ALL cases within Hispanic (*P* = 0.016, Fisher exact test) and Hispanic low-income subjects (*P* = 0.038) demonstrated significantly lower supplemental folate intake. High-income subjects, in contrast, demonstrated significantly higher intake of supplemental DFEs in ALL cases (*P* = 0.020). Across demographic subgroups (Supplementary Table S2), Hispanic subjects had significantly higher intake of food, fortified, and natural DFEs compared to non-Hispanic White subjects (Two sample Student’s *T* Test, *P* = 3.55 × 10^−5^, 0.015, and 9.28 × 10^−6^, respectively). Low-income subjects had significantly lower intake of total DFEs compared to high-income subjects (*P* = 0.016), despite also having higher intake of natural DFEs (*P* = 0.001). Hispanic subjects of low-income had significantly higher intake of food and natural DFEs (*P* = 6.48 × 10^−4^ and 1.93 × 10^−4^). Multi-variable linear regression analysis, controlling for ALL case status, sex, batch effect, nucleated cell composition (from the top 10 principal components derived from ReFACTor analysis) and ethnic structure (from the top 10 principal components derived from EPISTRUCTURE analysis), was completed on 700 754 CpGs passing QC measures. Linear regression was completed for DFEs independently for 5 dietary folate sources, including total DFEs, food-derived DFEs, fortified food DFEs, naturally occurring food DFEs, and supplemental folic acid DFEs. Comb-P was subsequently used to identify significant DMRs for meet-in-the-middle analysis, resulting in 30 significant DMRs for total DFEs, 32 for food DFEs, 28 for fortified DFEs, 32 for natural DFEs, and 35 for supplemental DFEs (Fig. 2).

**Table 2:** Folate distribution by demographic subgroup.

All subjects	All (*N* = 394)	Cases (*n* = 188)	Controls (*n* = 206)	*P*
Total DFEs	635.7 (391.9)	636.9 (388.3)	634.6 (396.1)	0.954
Food DFEs	480.9 (276.8)	480.8 (274.1)	480.9 (279.9)	0.999
Fortified DFEs	203.5 (193.9)	199.9 (194.2)	206.8 (194.0)	0.725
Natural DFEs	277.4 (154.3)	281.0 (156.0)	274.1 (153.0)	0.661
Supplemental DFEs				
0	281	134	147	0.893
194	27	12	15	
360	14	8	6	
540	72	34	38	
**Hispanic subjects**	**All (*N* = 174)**	**Cases (*n* = 82)**	**Controls (*n* = 92)**	***P***
Total DFEs	644.6 (397.0)	595.9 (370.7)	688.0 (416.4)	0.125
Food DFEs	549.2 (320.3)	547.4 (319.8)	550.7 (322.5)	0.945
Fortified DFEs	234.1 (217.4)	238.8 (221.0)	230.0 (215.2)	0.791
Natural DFEs	315.0 (179.0)	308.6 (177.7)	320.8 (180.9)	0.654
Supplemental DFEs				
0	142	75	70	0.016*
194	9	5	4	
360	4	2	2	
540	19	3	16	
**Non-Hispanic** **White subjects**	**All (*N* = 160)**	**Cases (*n* = 75)**	**Controls (*n* = 85)**	***P***
Total DFEs	697.9 (408.1)	697.9 (408.1)	575.9 (370.9)	0.051
Food DFEs	428.5 (208.5)	428.5 (208.5)	420.5 (226.8)	0.817
Fortified DFEs	168.2 (149.2)	168.2 (149.2)	194.7 (184.6)	0.316
Natural DFEs	260.3 (124.1)	260.3 (124.1)	225.7 (97.4)	0.055
Supplemental DFEs				
0	101	42	59	0.053
194	11	3	8	
360	7	4	3	
540	41	26	15	
**High-income** **subjects**	**All (*N* = 137)**	**Cases (*n* = 58)**	**Controls (*n* = 79)**	***P***
Total DFEs	701.9 (405.3)	766.0 (431.6)	654.8 (380.8)	0.12
Food DFEs	447.3 (260.8)	437.7 (243.4)	454.4 (274.2)	0.708
Fortified DFEs	201.1 (199.8)	190.7 (180.9)	208.8 (213.4)	0.593
Natural DFEs	246.2 (124.3)	247.0 (137.7)	245.6 (114.4)	0.949
Supplemental DFEs				
0	73	24	49	0.02*
*194*	15	6	9	
*360*	7	6	1	
540	42	22	20	
**Low-income** **subjects**	**All (*N* = 257)**	**Cases (*n* = 130)**	**Controls** (***n* = 127)**	***P***
Total DFEs	600.4 (380.7)	579.3 (354.1)	622.0 (406.3)	0.37
Food DFEs	498.7 (283.8)	500.1 (285.5)	497.4 (283.1)	0.939
Fortified DFEs	204.7 (199.1)	204.0 (200.5)	205.5 (181.9)	0.949
Natural DFEs	294.0 (165.9)	296.1 (161.7)	291.9 (170.8)	0.837
Supplemental DFEs				
0	208	110	98	0.379
194	12	6	6	
360	7	2	5	
540	30	12	18	
**Hispanic low-income subjects**	**All (*N* = 149)**	**Cases (*n* = 74)**	**Controls** (***n* = 75)**	***P***
Total DFEs	618.4 (381.0)	570.0 (336.5)	666.2 (417.0)	0.123
Food DFEs	549.3 (309.7)	543.7 (317.2)	554.8 (304.1)	0.8275
Fortified DFEs	229.7 (210.5)	235.3 (228.6)	224.4 (192.5)	0.7572
Natural DFEs	319.6 (178.1)	308.6 (168.2)	330.4 (187.8)	0.456
Supplemental DFEs				
0	129	69	60	0.038*
194	6	3	3	
360	2	0	2	
540	12	2	10	
**Non-Hispanic** **White low-income subjects**	**All (*N* = 78)**	**Cases (*n* = 41)**	**Controls (*n* = 37)**	***P***
Total DFEs	589.9 (392.3)	652.4 (404.6)	520.7 (371.4)	0.138
Food DFEs	424.3 (226.6)	453.3 (236.1)	392.1 (214.3)	0.233
Fortified DFEs	181.9 (164.1)	175.5 (154.5)	189.1 (176.0)	0.72
Natural DFEs	242.3 (125.3)	277.8 (145.2)	203.0 (84.5)	0.006**
Supplemental DFEs				
0	55	27	28	0.688
194	4	2	2	
360	4	2	2	
540	15	10	5	

Mean daily folate equivalent (DFE) levels assessed through the modified Block food frequency questionnaire are shown by folate source. Standard deviation is shown in parentheses. For Supplemental DFEs, subject counts for each intake level (0, 194, 360, 540) are shown. *P* values represent results of an two sample Student’s *T* test for continuous DFEs (Total, Food, Fortified, and Natural), and Fisher’s exact test for Supplemental DFEs.

* *P* < 0.05, ** *P* < 0.01.

### Meet-in-the-middle analysis

Overlapping genomic coordinates from leukemic blast DMRs and each of the five sources of folate DMRs were analyzed to identify regions of DNA methylation significantly associated with both leukemic blasts and periconceptional folate intake (Fig. 2). Since periconceptional folate is inversely associated with ALL, we would predict that DNA methylation impacts of periconceptional folate at birth would be *positively* associated with the same DNA methylation marks in the tumor cell (and vice versa) – should DNA methylation be a means by which periconceptional folate may directly mediate ALL risk by influencing methylation in the opposite direction. A total of 17 (56.7%) DMRs overlapped for total DFEs, 13 (40.6%) for food DFEs, 10 (35.7%) for fortified DFEs, 13 (40.6%) for natural DFEs, and 18 (51.4%) for supplemental DFEs (Table 3). To identify common directionality of effect across these overlapping regions, coefficients from leukemic blast Set A and Set B and the folate regression analysis were averaged across probes contained within each DMR, resulting in a positive or negative directionality for each region. Instances in which directionality differed between Set A and Set B were subsequently omitted. Direction of effect was equivalent between leukemic blast and folate in 7 DMRs for total DFEs (binomial test *P* = 0.629), 6 for food DFEs (*P* = 1), 3 for fortified DFEs (*P* = 0.344), 4 for natural DFEs (*P* = 0.267), and 16 for supplemental DFEs (*P* = 0.001). Notably, for supplemental DFE, the significant concordance in direction of effect was positive for both the leukemic blast and folate analyses (Table 4). Gene set enrichment analysis of overlapping regions did not identify significant association with gene ontology or KEGG pathway terms (Supplementary Tables S3, 4).

**Table 3:** Meet in the middle analysis overlapping regions.

	Concordant overlaps	Total overlaps	*P*
All subjects			
*Total DFE*	7	17	0.6291
*Food DFE*	6	13	1
*Fortified DFE*	3	10	0.3438
*Natural DFE*	4	13	0.2668
*Supplemental DFE*	16	18	0.001312**
Hispanic subjects			
*Total DFE*	2	3	1
*Food DFE*	4	9	1
*Fortified DFE*	4	6	0.6875
*Natural DFE*	2	6	0.6875
*Supplemental DFE*	7	12	0.7744
Non-Hispanic White subjects			
*Total DFE*	6	13	1
*Food DFE*	5	10	1
*Fortified DFE*	5	15	0.3018
*Natural DFE*	3	7	1
*Supplemental DFE*	4	11	0.5488
Low-Income subjects			
*Total DFE*	6	13	1
*Food DFE*	3	19	0.004425**
*Fortified DFE*	1	6	0.2188
*Natural DFE*	5	37	0.00000743***
*Supplemental DFE*	5	12	0.6072
High-income subjects			
*Total DFE*	4	12	0.3877
*Food DFE*	8	11	0.2266
*Fortified DFE*	4	9	1
*Natural DFE*	1	1	1
*Supplemental DFE*	4	7	1
Hispanic low-income subjects			
*Total DFE*	8	15	1
*Food DFE*	4	11	0.5488
*Fortified DFE*	3	4	0.625
*Natural DFE*	9	31	0.02945*
*Supplemental DFE*	10	19	1
Non-Hispanic White low-income subjects			
*Total DFE*	0	0	–
*Food DFE*	0	1	1
*Fortified DFE*	0	0	–
*Natural DFE*	0	2	0.5
*Supplemental DFE*	0	0	–

Total overlapping differentially methylated regions (DMRs) identified from the periconceptional folate epigenome-wide association study (EWAS) and acute lymphoblastic leukemia (ALL) EWAS are shown by dietary or supplemental source of folate. Concordant overlaps are those in which the mean direction of effect for the DMR (hypermethylated or hypomethylated) is consistent between the folate and ALL EWAS results. *P*-values for significant concordance or discordance in direction of effect obtained using a binomial test.

*DFE* dietary folate equivalent.

* *P* < 0.05, ** *P* < 0.01, *** *P* < 0.001.

**Table 4:** Overlapping differentially methylated regions (DMRs) for all subjects.

Chr	Gene name	Leukemic blast region				Folate region				Mean coefficient direction for region		
		Start Pos	End Pos	Probes	Šidák P	Start Pos	End Pos	Probes	Šidák P	Folate DFE	Blast Set A	Blast Set B
Total DFEs												
1	*LAX1*	203733971	203734559	5	1.06E−10	203734167	203734559	7	1.04E−04	−	−	−
1	*SLC35F3*	234366259	234367586	6	7.38E−08	234367322	234367586	4	0.04242	−	+	+
10	*GDF2*	48416463	48417904	11	1.38E−06	48416839	48416977	6	0.002751	−	+	+
1	*LAX1*	77417567	77417738	2	6.70E−04	77417567	77417738	2	0.01228	−	+	+
15	*UBE3A*	25683075	25685368	18	8.50E−30	25684818	25684849	3	0.0237	+	−	−
17	*C17orf98*	36997420	36998108	9	3.97E−05	36997420	36997740	8	0.001372	+	+	+
19	*EVI5L*	7926080	7928773	8	1.59E−04	7928546	7928773	3	6.10E−04	+	+	+
19	*SHANK1*	51170356	51172144	8	1.48E−06	51170137	51170356	3	0.01033	+	+	+
2	*MEIS1*	66671478	66673985	10	7.41E−26	66672299	66672553	3	0.03284	+	+	+
3	*TUSC4;CYB561D2*	50387146	50388910	18	2.44E−21	50388823	50388924	5	0.001263	+	+	+
6	*NA*	28583971	28584464	12	7.97E−14	28583971	28584121	9	0.02547	−	+	+
6	*RNF39*	30038720	30039600	27	7.59E−13	30039132	30039600	14	1.16E−05	−	+	+
6	*PPT2;PRRT1*	32115211	32123701	112	1.88E−27	32120584	32121475	27	4.54E−12	+	+	+
6	*PPP1R2P1*	32847530	32847845	10	8.15E−04	32847530	32847845	11	0.03056	−	+	+
6	*CRISP2*	49681178	49681391	7	1.51E−08	49681178	49681851	12	2.12E−07	−	+	+
7	*LOC100132707*	154719422	154720452	4	4.07E−43	154720213	154720452	3	0.009585	+	−	−
9	*C9orf135*	72435533	72436057	3	3.32E−07	72435785	72436057	3	3.87E−05	−	+	+
Food DFEs												
1	*LAX1*	203733971	203734559	5	1.06E−10	203734167	203734559	7	7.11E−07	−	−	−
11	*NADSYN1*	71209416	71210295	4	0.01948	71210210	71210295	2	0.00928	−	−	−
12	*KCNA6*	4916913	4919230	11	2.81E−06	4918075	4919230	10	6.74E−14	+	+	+
1	*LAX1*	1133074	1133378	5	5.28E−08	1133074	1133378	5	0.0123	−	+	+
2	*PLEKHH2;LOC728819*	43903227	43903842	5	3.52E−04	43903470	43904011	6	6.66E−06	−	+	+
2	*LOC100287216;SH3RF3*	109744523	109747264	17	1.01E−06	109746691	109747003	5	0.001069	−	+	+
3	*TUSC4;CYB561D2*	50387146	50388910	18	2.44E−21	50388823	50388924	5	9.69E−08	+	+	+
4	*EIF4E*	99849011	99851211	19	3.99E−06	99850801	99851211	8	1.42E−05	−	−	−
4	*TACR3*	104640197	104641548	11	3.86E−17	104640833	104641319	7	0.002247	−	+	+
6	*HLA-DQB2*	32728862	32730299	21	4.93E−11	32729442	32729876	13	3.53E−09	−	+	+
6	*SLC17A5*	74363018	74364700	10	1.32E−19	74364495	74364700	4	0.02705	−	−	−
7	*NA*	18125881	18127468	9	6.78E−06	18126902	18127112	4	0.02467	−	+	+
7	*LOC100132707*	154719422	154720452	4	4.07E−43	154719422	154720452	5	2.47E−08	+	−	−
Fortified DFEs												
10	*ZNF33B*	43133935	43134302	4	1.84E−08	43133935	43134302	5	0.002341	+	−	−
10	*CYP2E1*	135340445	135342218	12	0.004687	135340467	135340871	7	0.01295	−	+	+
11	*NADSYN1*	71209416	71210295	4	0.01948	71210210	71210295	2	0.03506	−	−	−
10	*ZNF33B*	93614970	93617168	19	1.29E−17	93616894	93617168	12	5.84E−04	+	−	−
19	*SPIB*	50931222	50931875	7	6.51E−05	50931222	50931622	6	2.58E−05	+	−	−
2	*PLEKHH2;LOC728819*	43903227	43903842	5	3.52E−04	43903470	43903842	5	0.003372	−	+	+
21	*NA*	45246252	45247038	5	1.48E−06	45246252	45246441	4	0.01049	+	+	+
6	*HLA-DQB2*	32728862	32730299	21	4.93E−11	32729442	32729876	13	3.71E−07	−	+	+
6	*C6orf122;C6orf208*	170190454	170191082	6	0.02188	170190914	170191082	4	0.01822	+	+	+
7	*HOXA4*	27167828	27171401	25	2.23E−07	27170552	27171213	12	0.001559	−	+	+
Natural DFEs												
1	*ISG15*	948740	948893	3	8.96E−35	948740	948893	5	9.35E−04	+	−	−
1	*LAX1*	203733971	203734559	5	1.06E−10	203734167	203734559	7	8.60E−06	−	−	−
10	*GRID1*	88022744	88023243	6	1.31E−07	88022859	88023185	4	0.01043	−	+	+
1	*ISG15*	4916913	4919230	11	2.81E−06	4918075	4919230	10	6.74E−14	+	+	+
12	*TMPRSS12*	51236582	51236768	6	5.42E−07	51236582	51236768	6	3.37E−05	−	+	+
12	*AGAP2;LOC100130776*	58119543	58121004	8	1.48E−12	58119915	58120237	6	0.007224	+	+	+
2	*C2orf84*	24396224	24398859	13	3.00E−09	24397539	24397871	7	0.01657	−	+	+
2	*LOC100287216;SH3RF3*	109744523	109747264	17	1.01E−06	109746691	109747003	5	0.006027	+	−	−
22	*FAM118A*	45703899	45706726	18	5.42E−13	45704902	45705042	6	0.01679	−	+	+
4	*EIF4E*	99849011	99851211	19	3.99E−06	99850801	99851211	8	2.33E−04	−	−	−
5	*BHMT2;DMGDH*	78365255	78366076	7	3.20E−10	78365647	78365830	7	9.77E−04	−	+	+
6	*NA*	28583971	28584464	12	7.97E−14	28583971	28584155	11	0.001066	−	+	+
6	*PPP1R2P1*	32847530	32847845	10	8.15E−04	32847530	32847845	11	0.009355	−	+	+
Supplemental DFEs												
10	*NA*	65732826	65733575	5	4.19E−04	65733273	65733575	4	0.02468	+	+	+
11	*CAT*	34460107	34461028	13	3.30E−22	34460107	34460557	11	3.66E−05	+	−	−
12	*PIWIL1*	130821453	130824831	18	8.92E−22	130824015	130824529	5	2.25E−04	+	+	+
10	*NA*	104551553	104552397	7	2.64E−16	104551481	104551553	3	0.00429	+	+	+
15	*MIR548H4;NOX5;SPESP1*	69221572	69223018	5	4.73E−07	69222895	69223018	5	9.18E−06	+	+	+
15	*ANKRD34C*	79574773	79576298	10	2.79E−10	79575334	79575634	2	0.02692	+	+	+
16	*ABAT*	8806359	8807043	12	9.74E−10	8806690	8807043	8	0.02621	−	+	+
18	*CABYR*	21718735	21719568	16	4.08E−06	21719352	21719568	6	1.76E−04	+	+	+
19	*SNAPC2*	7982713	7986206	14	3.86E−08	7983877	7984171	5	0.01084	+	+	+
20	*GNAS*	57425515	57428473	58	1.79E−06	57427412	57427977	18	7.22E−07	+	+	+
3	*NA*	127633887	127634587	6	1.54E−08	127634188	127634587	7	0.003615	+	+	+
3	*NA*	133502564	133503437	6	5.23E−06	133502564	133502952	8	1.51E−04	+	+	+
4	*NA*	77341251	77342788	7	9.51E−09	77341841	77342104	4	0.02735	+	+	+
6	*NA*	28601271	28601519	10	4.66E−11	28601271	28601519	10	0.006544	+	+	+
7	*FOXK1*	4762070	4763182	7	0.008631	4762236	4762371	2	9.11E−08	−	−	−
7	*ABCA13*	48493792	48494734	7	3.34E−13	48494362	48494734	5	5.35E−05	+	+	+
8	*BAI1*	143580770	143581070	3	0.04011	143580770	143581070	3	0.01311	+	+	+
8	*LYNX1*	143858414	143859990	18	1.18E−09	143859410	143860090	10	1.15E−13	+	+	+

Overlapping differentially methylated regions (DMRs) identified using Comb-P on epigenome-wide association study (EWAS) for periconceptional folate and acute lymphoblastic leukemia (ALL) DMRs are shown by dietary or supplemental source of folate. *Šidák P* represents *P*-value after adjustment for multiple comparisons across the genome.

*Chr* chromosome, *DFE* dietary folate equivalent, *Pos* Position.

### Subgroup analysis

Stratified assessment of Hispanic and non-Hispanic White participants demonstrates no significant directionality in overlapping regions (Supplementary Table S5, 6). In low annual income participants, there was a significant opposition in direction of effect for food DFEs (3 of 19 overlapping DMRs, *P* = 0.004) and natural DFEs (5 of 37 overlapping DMRs, *P* = 7.43 × 10^−6^, Table 5, Supplementary Table S7), while there was no significant directionality identified in high annual income participants (Supplementary Table S8). In Hispanic participants of low annual income, a significant opposition in direction of effect was again seen for natural DFEs (9 of 31 overlapping DMRs, *P* = 0.029, Table 5, Supplementary Table S9), while no significant directionality was seen in overlaps for non-Hispanic White participants of low annual income (Supplementary Table S10).

**Table 5:** Overlapping regions between folate and leukemic blast analyses by Hispanic ethnicity and income status subsets with significantly discordant for direction of effect.

Chr	Gene name	Leukemic blast region				Folate region				Mean coefficient for region		
		Start Pos	End Pos	Probes	Šidák P	Start Pos	End Pos	Probes	Šidák P	Folate DFE	Blast Set A	Blast Set B
Low income subjects												
Food DFEs												
1	*NA*	247802703	247803166	7	7.16E−08	247802703	247803033	7	0.01065	−	+	+
10	*PAOX*	135202522	135203200	5	3.38E−06	135202522	135203200	7	1.21E−08	+	−	−
1	*NA*	102702285	102702656	4	1.81E−10	102702414	102702656	3	2.20E−06	−	+	+
12	*TMPRSS12*	51236582	51236768	6	5.42E−07	51236446	51236768	7	0.01819	−	+	+
13	*NA*	76444798	76445108	7	0.03747	76444798	76445108	8	0.03423	−	+	+
14	*DIO2*	80676274	80677688	4	1.65E−04	80677608	80677847	4	2.34E−04	−	+	+
14	*MEG3*	101290195	101294430	29	2.23E−07	101294430	101294623	2	0.01108	−	+	+
16	*ANKRD26P1*	46603015	46604297	7	4.23E−12	46603015	46603321	6	0.03711	−	+	+
19	*C19orf6*	1008643	1009949	4	0.001138	1009642	1009949	5	0.003159	−	+	+
2	*C2orf65*	74875253	74876010	8	2.00E−15	74875253	74875561	8	0.008844	−	+	+
2	*LOC100287216;SH3RF3*	109744523	109747264	17	1.01E−06	109746691	109747003	5	0.002443	−	+	+
6	*MAS1L*	29454623	29455256	7	0.008821	29454623	29454954	6	1.37E−05	−	+	+
6	*HLA-DQB2*	32728862	32730299	21	4.93E−11	32729442	32729876	13	1.19E−04	−	+	+
6	*PPP1R2P1*	32847530	32847845	10	8.15E−04	32847530	32847845	11	0.03313	−	+	+
7	*PON3*	95025194	95027158	24	8.16E−15	95025855	95026211	13	6.53E−04	−	+	+
8	*SCARA5*	27737078	27737223	2	3.01E−04	27737078	27737223	2	0.02614	−	+	+
Natural DFEs												
1	*GTF2B*	89356964	89358043	8	4.86E−21	89357908	89358043	5	0.02563	−	+	+
1	*NA*	117317838	117318232	5	0.01862	117317903	117318232	4	0.009964	−	+	+
1	*NA*	242220538	242220925	3	8.03E−04	242220538	242220925	3	4.56E−05	−	+	+
1	*GTF2B*	88022744	88023243	6	1.31E−07	88022744	88023243	6	4.86E−10	−	+	+
10	*SLC16A12*	91295159	91296457	14	7.35E−16	91296252	91296457	3	0.03084	−	+	+
10	*PAOX*	135202522	135203200	5	3.38E−06	135202611	135203102	5	0.02039	+	−	−
13	*NA*	21900506	21901649	4	3.67E−05	21900392	21900810	4	0.00105	−	+	+
13	*TRPC4*	38443634	38445267	12	5.54E−16	38445013	38445267	2	0.01753	−	+	+
14	*DIO2*	80676274	80677688	4	1.65E−04	80677608	80677847	4	0.001305	−	+	+
19	*PPP1R13L*	45884900	45885922	3	3.66E−09	45885800	45886078	4	0.01325	−	+	+
2	*C2orf84*	24396224	24398859	13	3.00E−09	24397539	24397871	8	1.58E−05	−	+	+
2	*GTF2A1L;STON1-GTF2A1L*	48844728	48845068	7	1.40E−07	48844728	48845068	7	0.002962	−	+	+
2	*BOLL*	198649771	198651576	16	5.23E−23	198649767	198650112	6	9.40E−04	−	+	+
2	*ANKMY1*	241458886	241460664	8	1.27E−11	241458886	241459227	4	0.00544	−	+	+
20	*BLCAP;NNAT*	36147042	36150061	40	9.98E−06	36148375	36149271	31	7.56E−06	−	+	+
3	*PRR23A*	138725153	138725425	7	1.14E−14	138725153	138725425	8	4.33E−04	−	+	+
4	*RBM46*	155702172	155703429	11	3.57E−06	155702760	155703429	5	1.92E−04	−	+	+
5	*NA*	131281008	131281574	6	0.004398	131281008	131281439	6	1.43E−04	−	+	+
6	*NA*	28583971	28584464	12	7.97E−14	28583971	28584155	11	3.33E−04	−	+	+
6	*NA*	28601271	28601519	10	4.66E−11	28601271	28601519	10	9.98E−04	−	+	+
6	*NA*	28828946	28832672	50	1.06E−14	28829171	28829640	10	2.86E−08	−	+	+
6	*TNXB*	32063394	32066121	42	4.45E−04	32063835	32064258	15	0.001266	−	+	+
6	*HLA-DQB2*	32728862	32730299	21	4.93E−11	32729442	32729823	12	0.005971	−	+	+
6	*PPP1R2P1*	32847530	32847845	10	8.15E−04	32847530	32847845	11	0.01474	−	+	+
6	*CRISP2*	49681178	49681391	7	1.51E−08	49681178	49681851	12	2.68E−09	−	+	+
6	*SPACA1*	88757302	88757878	6	5.18E−07	88757302	88757392	5	0.006156	−	+	+
6	*NA*	112688010	112688930	4	0.03221	112688010	112688607	3	0.002751	−	+	+
6	*C6orf174*	127796287	127797286	7	1.71E−07	127796989	127797286	4	0.01374	−	+	+
7	*PON3*	95025194	95027158	24	8.16E−15	95025836	95026248	15	1.75E−04	−	+	+
7	*REPIN1*	150067807	150069890	7	2.33E−07	150069577	150069890	5	0.04007	−	+	+
7	*NA*	156400281	156400990	5	8.23E−05	156400711	156400990	5	0.01408	−	+	+
X	*FAM46D*	79590789	79591032	8	1.96E−08	79590789	79591032	8	2.42E−04	−	+	+
Hispanic low income subjects												
Natural DFEs												
1	*NAV1*	201708522	201709675	9	1.16E−09	201708419	201708767	6	1.11E−04	+	−	−
1	*MFSD4*	205560943	205561906	11	1.48E−23	205561090	205561401	6	0.00579	+	−	−
1	*NA*	242220538	242220925	3	8.03E−04	242220538	242220925	3	1.07E−04	−	+	+
1	*NAV1;NAV1*	88022744	88023243	6	1.31E−07	88022859	88023243	5	1.70E−04	−	+	+
15	*LASS3*	101084507	101085710	8	1.14E−16	101084980	101085388	8	7.31E−04	−	+	+
16	*UMOD;UMOD*	20359444	20361044	8	8.52E−06	20360206	20360497	4	0.001706	−	+	+
17	*WFIKKN2*	48911141	48913424	21	7.52E−05	48912164	48912860	13	2.06E−06	−	+	+
19	*LYPD3;LYPD3*	43969650	43970683	8	7.96E−04	43969650	43970048	7	5.29E−05	−	+	+
19	*LYPD5*	44324467	44325008	11	1.02E−18	44324747	44325139	10	6.91E−04	−	+	+
19	*PPP1R13L*	45884900	45885922	3	3.66E−09	45885800	45886078	4	2.53E−05	−	+	+
2	*GTF2A1L;STON1-GTF2A1L*	48844728	48845068	7	1.40E−07	48844728	48845068	7	4.39E−06	−	+	+
20	*BLCAP;NNAT*	36147042	36150061	40	9.98E−06	36148375	36149231	30	4.08E−06	−	+	+
22	*FAM118A*	45703899	45706726	18	5.42E−13	45704675	45705265	8	2.87E−17	−	+	+
3	*MAGI1*	65342216	65342971	5	5.21E−14	65342546	65342843	3	0.01418	−	+	+
3	*PRR23A*	138725153	138725425	7	1.14E−14	138725153	138725425	8	0.007163	−	+	+
4	*RBM46*	155702172	155703429	11	3.57E−06	155702409	155703429	9	1.38E−07	−	+	+
4	*ODZ3*	183721183	183721778	5	1.19E−04	183721565	183721778	4	0.02955	−	+	+
6	*NA*	28828946	28832672	50	1.06E−14	28829171	28829640	10	1.62E−05	−	+	+
6	*TNXB*	32063394	32066121	42	4.45E−04	32063595	32064956	37	2.86E−13	−	+	+
6	*NA*	90596146	90597591	9	5.81E−04	90597340	90597591	4	0.007835	−	+	+
7	*NA*	156400281	156400990	5	8.23E−05	156400711	156400990	5	0.02597	−	+	+
9	*NA*	90439478	90440066	5	6.88E−09	90439478	90439702	3	0.02084	−	+	+

Only regions discordant between folate and leukemic blast analyses are shown. *Šidák* *P* represents *P*-value after correction for multiple comparisons across the genome.

*Chr* chromosome, *DFE* dietary folate equivalent, *Pos* Position.

## Discussion

These results identify overlapping regions of DNA methylation responsive to periconceptional folate at birth and within leukemic blasts at the time of pediatric ALL diagnosis. This includes 17 overlapping DMRs for total folate, 13 for food, 10 for fortified, 13 for natural, and 18 for supplemental folate intake. These findings indicate that sites of DNA methylation susceptible to periconceptional folate intake evident in a polyclonal birth blood cellular population are additionally associated with DNA methylation variation in leukemic cells proliferating from monoclonal precursors months to years later at the time of diagnosis at rates ranging from 35.7% to 56.7% by folate source. This implies the association between maternal folate intake and pediatric ALL risk may in part be reflected through alterations in DNA methylation identifiable at birth.

Interestingly, overlapping regions associated with dietary sources of folate (i.e. fortified and natural categories) showed a non-significant bias in opposing the direction of effect to that seen in leukemic blasts. This would indicate early variation in DNA methylation in response to dietary folate is driven in the opposite direction as that seen in leukemic blasts at diagnosis (i.e., hypomethylated at birth at sites found to be hypermethylated in leukemic blasts) and support a plausible *protective mechanism* for dietary folate. In addition, given the association between maternal dietary quality and reduction in pediatric ALL [5, 38], our findings related to dietary sources of folate may further reflect a biological mediation or manifestation of the beneficial effect of healthier diet on childhood leukemia risk.

These effects further varied by Hispanic ethnicity and income. While children with low annual income experienced opposing direction of effect for food and natural folate, there was no significant relationship with supplemental folate (i.e., vitamin pills). While there were no concordance or discordance in direction of effect in Hispanic or non-Hispanic White participants as a whole, Hispanic participants of low annual income presented a significant *opposition* in deflection of effect compared to natural folate. Here, while total folate intake was lower in Hispanic subjects, folate derived from food sources (both fortified and natural) was significantly higher in this group compared to non-Hispanic White subjects, who derived a larger proportion of total folate intake from supplements. Increased folate intake from natural sources may indicate an overall higher dietary quality, however this relationship is impacted by multiple cultural and socioeconomic factors within Hispanic subjects in California [39]. We previously demonstrated variation in the relationship between periconceptional folate and DNA methylation at birth by Hispanic ethnicity, annual income and education level [22] – specifically, these subgroups showed a higher magnitude of associations with supplemental folate. Our findings indicate that Hispanic children may benefit specifically from increased dietary folate in the pre/periconceptional period, and that this mechanism may in part be driven through early modifications in DNA methylation patterns. Notably, compared to all other racial/ethnic groups in the United States, Hispanic children have the highest risk of ALL [40], in addition to neural tube defects which are an additional folate-sensitive developmental morbidity [41].

In contrast to dietary folate, supplemental folate was significantly more likely to share a concordant direction of effect in overlapping regions to that of leukemic blasts. These results would imply folic acid supplementation drives DNA methylation toward the state seen in ALL at diagnosis. There are multiple potential explanations for the difference seen for dietary vs. supplemental folate. The bioavailability of naturally occurring folates in food is ~80% of that from folic acid containing supplements [42], presumably resulting in greater levels of folate available to the developing fetus. The availability of naturally occurring folates may be further reduced based on food handling and cooking practice. Thus, total folate exposure to the fetus from folic acid supplementation likely exceeds that of folate from food for the same measured intake from our survey data, which may impact the manner in which DNA methylation patterns are established. Similarly, the balance of other nutrients in food are likely to be highly varied in comparison to those of pre-natal and multi-vitamin supplements. Together, this would suggest potential variation in the mechanism by which folate impacts the early stages of ALL pathogenesis by folates found in food compared to folic acid from dietary supplements, and that excessively high doses of folic acid may contribute toward leukemia development. This finding would seem intriguing given Sydney Farber’s work demonstrating that folic acid accelerates the progression of acute leukemia [43], establishing anti-folates as the basis of early ALL treatment approaches [44]. However, epidemiological studies would contradict this premise, as folic acid supplementation shares a similar protective effect to that seen in dietary folate [15]. This relationship may further represent more complex gene-nutrient interactions in folate replete and depleted states [45], as higher supplemental folate levels generally coincided with higher total folate intake in our cohort. Variants in folate pathway genes leading to higher predicted serum folate levels are associated with reduced risk of ALL [14] – while dietary folate intake appears to mitigate this effect in Hispanic subjects, intake in Non-Hispanic White subjects did not alter their genetically determined risk of ALL. Notably, the mBFFQ assessment of supplements did not specifically distinguish between folic acid and 5-methyltetrahydrofolate containing products, however we anticipate relatively minimal contribution of the latter to the supplemental DFE variable since it was introduced to vital supplements after the majority of our study population was enrolled.

This study benefitted from a large sampling of participants from the diverse population in California with available perinatal DNA methylation data and detailed maternal periconceptional dietary and supplement calculations. We did not specifically control for genetic influence on DNA methylation patterns, or methylation quantitative trait loci (mQTL). However, none of the top overlapping regions (Tables 4 and 5) contained CpGs with known mQTL effect identified in prior investigations [46], reducing the likelihood of genetic influence confounding interpretation of our results. A limitation of this study is the reliance on retrospective mBFFQ survey in the assessment of folate intake, for which no formal assessment of reliability exists [5]. However, in this instance the risk of bias is reduced given knowledge of the relationship between folate and ALL risk is less widely known in the general population, which may serve to limit variation in recall between cases and controls [47]. Additionally, direct measurement of maternal folate may vary temporally [48] and may not be fully reflective of maternal stores accessible to the developing early fetus. In addition, our analysis of ALL leukemic blasts did not utilize deconvolution to control for the impact of normal bone marrow nucleated cells. However, the majority of cells in the diagnostic samples used will be made up of leukemic blasts, and the impact of any remaining non-leukemic mononucleated cells is unlikely to substantially impact our results, which is consistent with previous studies of similar design [37, 49, 50]. Our analysis did not specifically account for other types of vitamin intake which may influence ALL risk, and thus it is possible that some degree of the differences we identified between total, food and supplemental folate intake may represent more complex interactions between dietary quality and nutrient intake.

In summary, we identified linkage between regions of DNA methylation responsive to periconceptional folate and differentially methylated in lymphoblasts at the time of pediatric ALL diagnosis. These associations vary in number and epigenomic region depending on dietary source. The direction of effect in DNA methylation variation associated with folic acid from food and, in particular, naturally occurring folate in dietary sources had an inverse relationship to that of lymphoblasts for Hispanic participants of low annual income. These results suggest a unique benefit to increased dietary folate in Hispanic population as a means to reduce incidence of pediatric ALL. Given reported associations between maternal genetic variants in folate pathway genes [13] and ALL risk, these findings call for further investigation of the relationship between periconceptional folate, genetic variants and DNA methylation as it relates to pediatric ALL risk.

## Supplementary information

Supplemental Tables

## Data Availability

All computer code generated for this analysis is available upon request from the authors. This study used biospecimens from the California Biobank Program, which prohibits uploading of genomic data (including genome-wide DNA methylation) and/or sharing of individual-level data obtained from these biospecimens under the statutory scheme of the California Health and Safety Code sections 124980(j), 124991(b) and (h), and 103850(a) and (d). Processed data files, including results from epigenome-wide association studies included in this manuscript are available as supplemental files.

## Code availability

All computer code generated for this analysis is available upon request from the authors.
